# Promotion of the Transition of Adult Patients with Childhood-Onset Chronic Diseases among Pediatricians in Japan

**DOI:** 10.3389/fped.2016.00111

**Published:** 2016-10-18

**Authors:** Yuko Ishizaki, Hirohiko Higashino, Kazunari Kaneko

**Affiliations:** ^1^Department of Pediatrics, Kansai Medical University, Hirakata, Osaka, Japan; ^2^Research Committee on the Investigation and Refined Policy to Support Social, Medical and Educational Life of Children with Chronic Diseases, Ministry of Health, Labour and Welfare of Japan, Tokyo, Japan; ^3^Higashino Clinic, Higashi-Osaka, Japan

**Keywords:** health-care transition, childhood-onset chronic diseases, pediatrician, promotion, Japan

## Abstract

The transition of adult patients with childhood-onset chronic diseases (APCCD) from pediatric to adult health-care systems has recently received worldwide attention. However, Japan is lagging behind European countries and North America as this concept of health-care transition was introduced only 10 years ago. In Japan, before the introduction of this concept, APCCD were referred to as “carryover patients,” who were often considered a burden in pediatric practice. In the late 1990s, groups composed of pediatric nephrologists, developmental and behavioral pediatricians, pediatric nurses, and special education teachers researching the quality of life of adult patients with chronic kidney disease began to discuss the physical and psychosocial problems of APCCD. In 2006, a group of pediatricians first introduced the term “transition” in a Japanese journal. By 2010, a group of adolescent nurses had begun a specialized training program aimed at supporting patients during the transitional period. In 2013, the Ministry of Health, Labour and Welfare in Japan convened a research committee, focusing on issues related to social, educational, and medical support for APCCD, and the Japan Pediatric Society established a committee for the health-care transition of APCCD and summarized their statements. Moreover, in 2013, the Tokyo Metropolitan Children’s Medical Center initiated ambulatory services for APCCD managed by specialized nurses. The concept of health-care transition has rapidly spread over these past 10 years. The purpose of this article is to describe how this concept of health-care transition has advanced in Japan, such that APCCD now experience a positive pediatric to adult health-care transition.

## Introduction

### Increase of Adult Patients with Childhood-Onset Chronic Disease

Advances in medical science have enabled many children with chronic diseases to survive till adulthood, often without serious sequelae or disabilities (1–3). These high survival rates have increased the number of adolescents transitioning from pediatric to adult health-care systems. In addition, the prevalence of certain chronic illnesses of childhood is increasing (4). These adult patients with childhood-onset chronic disease (APCCD) often have limited social experiences because of their childhood disease and have difficulties in adapting to adult social lives within their community and health-care systems (5).

In Japan, medical aid for specific chronic pediatric diseases (MASCPD) pays the medical fees for 514 kinds of intractable diseases that the Ministry of Health, Labour and Welfare of Japan (the Ministry) classified as requiring advanced medical treatment.^1^ Among APCCD registered in the MASCPD, 53.6% have complications due to their original diseases and 30.3% have experienced progression of their original diseases. With respect to the educational and socioeconomic status of APCCD, 55.3% of healthy individuals are college graduates compared with ≤40% or fewer of APCCD (6). With regard to the working status, 56.3% of APCCD report chronic illness to be a barrier in employment (6). The medical fees for APCCD are approximately four and five times higher than the national average in the 20- to 24-year and 30- to 34-year age groups, respectively. Therefore, programs are required to ensure a seamless transition from medical care in childhood and adolescence to that in adulthood and to help children grow socially and become independent, working adults.

### Before the Era of the Term “Transition” in Japan

The transition of APCCD from pediatric to adult health-care systems has recently received significant attention in literature worldwide (7–11). Transition has been defined as a multifaceted, active process that attends to the medical, psychosocial, and educational needs of adolescents as they move from pediatric to adult health-care systems (4). Although the importance of health care during this transition period has been acknowledged in Europe, the United States, and Australia,^2^^,^^3^^,^^4^ Japan lags behind. In Japan, before the term “healthcare transition” was introduced in 2006, APCCD >20 years who had continued to seek medical health-care from a pediatric service instead of switching to adult health-care providers were termed as “carryover” patients, and they were considered a burden in pediatric practice (12).

Adult patients with childhood-onset chronic diseases can continue their treatment with the pediatric health-care providers because of specifications within Japan’s health insurance system. In Japan, Japanese nationals, including those who are unemployed, can have their medical treatment fees reimbursed by the national health insurance system.^5^ Whether APCCD are treated by pediatric or adult health-care providers, their medical fees are paid by the national health insurance system; therefore, they do not realize the necessity of transition. However, a number of problems can result from APCCD choosing to continue treatment from pediatric providers (12).

## Previous Studies on APCCD in Japan

### From a Burden of Pediatricians Called as “Carryover” to Health-Care Transition

As a history of spreading the term “transition” in Japan, in the late 1990s, groups researching quality of life of adult patients with chronic kidney disease, comprising pediatric nephrologists, developmental and behavioral pediatricians, pediatric nurses, and special education teachers, have begun to discuss physical and psychosocial problems of APCCD. Then, a study of health-care problems of “carryover” patients was done, and the term “transition” was introduced at the same time in 2006 (12). In the study, we performed a qualitative analysis using medical records of APCCD within the disciplines of pediatric neurology, nephrology, hematology, and endocrinology to assess physical and psychosocial problems faced by “carryover” patients. In addition, we surveyed specialists for their opinions on whether they hoped to transfer “carryover” patients to adult health-care services and the perceived barriers in the transfer. The problems associated with “carryover” patients are summarized as follows (Table 1): pediatricians are not equipped to be the primary care provider for adult-onset diseases, such as malignant tumor, ischemic heart disease, and pregnancy. Adult patients feel uncomfortable within the pediatric environment, and they cannot be hospitalized in a pediatric ward. A common reported barrier of transfer is the strong confidential relationship between a guardian and the pediatrician. Pertaining to certain childhood-onset diseases, there are no relevant specialists in the adult health-care systems. Psychosocial problems related to APCCD, such as immature social development or mood disturbances, may inhibit the transfer of the patient.

**Table 1:** **Health-care problems of adult patients with child-onset chronic diseases (APCCD) in Japan (12)**.

Common problems in the assessment of adult patients by pediatricians
Problems that pediatricians cannot manage
Primary care of adult-onset diseases
Pregnancy and childbirth
Problems that an adult patient has
Feeling of uncomfortableness with the pediatric environment
Difficulty of hospitalized in a pediatric ward
Common factors that hinder the transfer
The strong relationship between guardian and pediatrician
Lack of APCCD specialists in adult health-care systems
Mental problems of APCCD

Although this was most probably the first study conducted in Japan on this subject, the results are similar to those of studies conducted overseas. On the basis of the results of this study, APCCD physical health and psychological independence were deemed important, and transfer of APCCD to an adult health-care system was believed to be of great benefit (12).

### Comparison of the Perception of Japanese Pediatricians and Child Health Nurses

We conducted a questionnaire survey of Japanese pediatricians and child health nurses regarding their perceptions of transition, and awareness of health-care problems of APCCD and “transition” programs was compared between pediatricians and nurses (13). The findings of the study were summarized: three-quarters of the pediatricians and all of the nurses reported that transition programs were necessary, a higher proportion of the nurses realized the necessity of transition and were aware that such programs had already been developed, and both pediatricians and nurses reported that the present network was not yet fully equipped and should, therefore, be expanded and bolstered with the involvement of pediatricians nationwide. The concepts of health-care transition had already progressed in nursing in Japan, well before pediatricians recognized its importance.

## Recent Advances in Health-Care Transition in Japan

### Beginning of the Usage and Promotion of Transition in Japan

**Figure 1:**
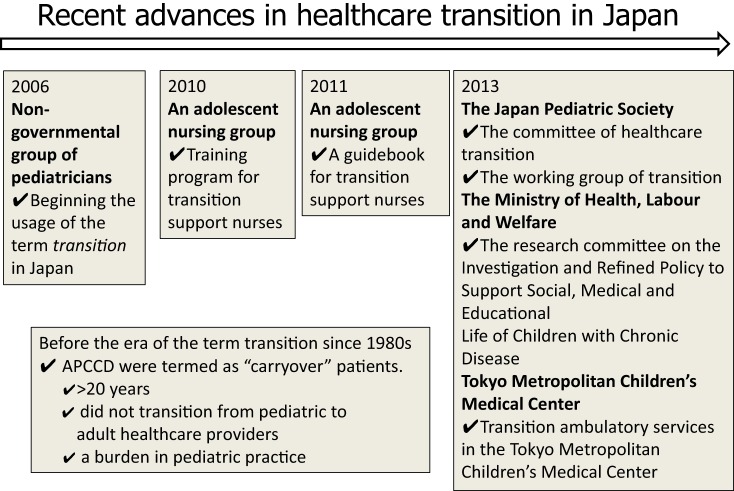
**History of promotion of the health-care transition of adult patients with child-onset chronic diseases (APCCD) in Japan**.

Recent advances in health-care transition in Japan are summarized in Figure 1. At the same time, we introduced the term “healthcare transition” to Japanese people; an adolescent nursing group introduced the concept of health-care transition to the adolescent nursing field as a whole. They instituted a specialized health-care transition nursing training program in 2010 and published a practical guidebook for health-care transition in Japan in 2011 (14).

In 2013, the first transition ambulatory service managed by specialized nurses was established in Tokyo Metropolitan Children’s Medical Center (15). In preparation, discussion on whether transitional health care is necessary for APCCD began in 2005, prior to the reconstruction of three children’s hospitals in Metropolitan Tokyo. It was concluded that health-care transition was indispensable. Subsequently, to prepare for transition, the followings were undertaken: lectures on health-care transition, trial use of the support guidebook for transition, and implementation of a transition working group since 2010. Finally, ambulatory services started in 2013.

In the field of pediatric nephrology that is a precursor of health-care transition of Japan, the Japanese Society of Nephrology (JSN) and the Japanese Society for Pediatric Nephrology (JSPN) cooperated and announced the statement of health-care transition of pediatric patients with chronic kidney diseases granted by Health and Labour Sciences Research Grants from the Ministry of Health, Labour and Welfare of Japan and published the statement in both JSN and JSPN official journal in 2015 (16, 17).

### Activities of the Japan Pediatric Society and the Ministry of Health, Labour and Welfare of Japan

The Japan Pediatric Society convened a committee of healthcare transition and summarized their statements in 2013, and the working group launched its activities in November 2013. In the statement of the committee of health care transition, the Japan Pediatric Society proposed they called APCCD “transitional period patients” and decided to no longer use the term “carryover” patients (18).

In 2012, the Ministry appointed a Research Committee on the Investigation and Refined Policy to Support Social, Medical, and Educational Life of Children with Chronic Disease, which became active in April 2013. One of the Research Committee’s activities was to develop a support guidebook on the transition of APCCD for pediatricians (15). Next to compiling the guidebook, subcommittees of the Japan Pediatric Society were surveyed regarding the preparation for and current status of transition. A questionnaire was distributed to 17 subcommittees of the Japan Pediatric Society and returned from 13 subcommittees (19). As a results of the study, all of the 13 subcommittees answered that there were candidates for transition to adult health-care systems within their pediatric specialties. However, only six subcommittees had working groups for transition, and only four groups had a guideline for transition within their disease specialty. Based on these findings, it was revealed that although subcommittees believed transition support to be necessary in their field, they are yet to fully develop and implement working groups and detailed guidelines.

### Limitations and Challenges in Implementing a Health-Care Transition

The type of health-care transition described here is only available for APCCD without severe physical or intellectual disabilities. Therefore, other health-care transition methods should be developed for APCCD with severe intellectual or physical disabilities.

Challenges include changing pediatricians’ beliefs that they can or must entirely support the physical and mental health of their pediatric patients. To address this challenge, we invited specialists, including nurse practitioners, medical social workers, and APCCD, to deliver lectures on the benefits of a successful health-care transition at seminars for pediatricians. However, we must develop additional opportunities to discuss the concept of health-care transition to achieve successful patient outcomes in the future.

## Summary

In Japan, pediatricians and child health nurses at clinical sites were the first to acknowledge and promote health-care transition from the pediatric to adult health-care system. In 2006, a group of pediatricians first introduced the term “transition” in a Japanese journal. Concurrently, a group of adolescent nurses began a specialized training program aimed at supporting patients during the transitional period and in 2010, published a practical guidebook for health-care transition.

From 2010s, the Japan Pediatric Society and Ministry of Health, Labour and Welfare have begun to take measures of their own and urge pediatricians, adult health providers, and adolescent medical practitioners to help promote transition. Concept of health-care transition has spread rapidly for these 10 years. Going forward, pediatric practitioners, the Japan Pediatric Society, and Ministry of Health, Labour and Welfare will cooperate to promote smooth transition from pediatric to adult health-care systems in Japan.

## Author Contributions

YI, HH, and KK participated in the design of this study. YI is a grant holder. YI and HH drafted the manuscript. KK supervised the manuscript. All authors read and approved the final version of this manuscript.

## Conflict of Interest Statement

The authors declare that this research was performed in the absence of any commercial or financial relationships that could be constructed as a potential conflict of interest.

## Footnotes

^1^Ministry of Health, Labour and Welfare of Japan. *Medical Aid for Specific Chronic Pediatric Diseases*. Available from: http://www.mhlw.go.jp/stf/seisakunitsuite/bunya/0000078973.html

^2^Bronheim S, Fiel S, Schidlow D, MaGrab P, Boczar K, Dillon C. *Crossing: A Manual for Transition of Chronically Ill Youth to Adult Health Care*. Available from: http://hctransitions.ichp.edu/CrossingsPDFs/Crossings.pdf

^3^Royal College of Nursing. *Adolescent Transitional Care Guidance for Nursing Staff*. Available from: https://www.rcn.org.uk/professional-development/publications/pub-004510

^4^NSW Agency for Clinical innovation. Available from: https://www.aci.health.nsw.gov.au/networks/transition-care

^5^Ministry of Health, Labour and Welfare of Japan. *National Health Insurance System*. Available from: http://www.mhlw.go.jp/stf/seisakunitsuite/bunya/kenkou_iryou/iryouhoken/iryouhoken01/index.html
